# Development of a Low-Cost Narrow Band Multispectral Imaging System Coupled with Chemometric Analysis for Rapid Detection of Rice False Smut in Rice Seed

**DOI:** 10.3390/s20041209

**Published:** 2020-02-22

**Authors:** Haiyong Weng, Ya Tian, Na Wu, Xiaoling Li, Biyun Yang, Yiping Huang, Dapeng Ye, Renye Wu

**Affiliations:** 1 College of Mechanical and Electrical Engineering, Fujian Agriculture and Forestry University, Fuzhou 310002, China; hyweng@zju.edu.cn (H.W.); 13133969161@163.com (Y.T.); yangbiyun2010@126.com (B.Y.); wing361686@gmail.com (Y.H.); 2 College of Biosystems Engineering and Food Science, Zhejiang University, Hangzhou 310058, China; nawu018@zju.edu.cn; 3 College of Mechanical Engineering and Automation, Fuzhou University, Fuzhou 310002, China; xiaoling_fzu@163.com; 4 College of Agriculture, Fujian Agriculture and Forestry University, Fuzhou 310002, China

**Keywords:** rice seed, rice false smut (RFS), multispectral imaging, least squares-support vector machine (LS-SVM), narrow band

## Abstract

Spectral imaging is a promising technique for detecting the quality of rice seeds. However, the high cost of the system has limited it to more practical applications. The study was aimed to develop a low-cost narrow band multispectral imaging system for detecting rice false smut (RFS) in rice seeds. Two different cultivars of rice seeds were artificially inoculated with RFS. Results have demonstrated that spectral features at 460, 520, 660, 740, 850, and 940 nm were well linked to the RFS. It achieved an overall accuracy of 98.7% with a false negative rate of 3.2% for *Zheliang*, and 91.4% with 6.7% for *Xiushui,* respectively, using the least squares-support vector machine. Moreover, the robustness of the model was validated through transferring the model of *Zheliang* to *Xiushui* with the overall accuracy of 90.3% and false negative rate of 7.8%. These results demonstrate the feasibility of the developed system for RFS identification with a low detecting cost.

## 1. Introduction

Rice false smut (RFS), also called as pseudo-smut, is caused by a pathogenic ascomycete fungus named *Ustilaginoidea virens* (*U. virens*) *Takah (teleomorph Villosiclava virens*), which can attack the stamen filaments of rice at the booting stage [1,2]. Apart from rice, maize, *Digitaria marginata, Imperata cylindrica*, and *Panicum trypheron* are also alternative hosts of *U. virens* [3]. It can produce both asexual (chlamydospores) and sexual (ascospores) stages during its life cycle [4]. It has spread to many rice-growing countries and become one of the most severe grain diseases, resulting in significant yield loss and contamination of grains and straws with ustiloxins [5,6]. Consequently, there is a need for rice false smut diagnosis methods that are accurate, rapid, and inexpensive. Currently, the initial screening is performed according to experienced experts, but it is a subjective assessment. The molecular identification based on polymerase chain reaction (PCR) is the most commonly used method for *U. virens* detection with high sensitivity and accuracy [7]. However, it is time-consuming, labor-intensive and expensive with highly professional skills required. 

Developing a rapid and non-destructive technique for the quality evaluation of agricultural plant seeds has attracted a great interest in different research backgrounds such as variety identification in maize seeds [8,9]; germination determination in tomato (Solanum lycopersicum L.) seeds [10]; and storage age discrimination in paddy seeds [11]. The reported study illuminated that short wave infrared hyperspectral imaging combined with chemometrics presented the potential for the rapid assessment of corn seed viability with up to 100% classification accuracy using the support vector machine (SVM) model [12]. The investigation on viability and vigor in muskmelon seeds was also carried out using near infrared hyperspectral imaging with the overall accuracy of 94.6% based on partial least-squares discriminant analysis [13]. Spectral bands related to green mottle mosaic virus infection in watermelon seeds were also investigated [14]. It was observed that the reflectance at 1411, 1456, 1792, and 1867 nm could be considered to detect virus-infected watermelon seeds with the overall detection accuracy of 83.3%. The research was also carried out to detect ochratoxin A (OTA) contaminated stored wheat in a stored period using line-scan near-infrared hyperspectral imaging with more than 98% classification accuracy using the reflectance at 1280, 1300, 1350, and 1480 nm [15]. Wu et al. 2020 proposed an effective method to detect rice false smut based on near-infrared hyperspectral imaging (NIR-HIS) combined with multivariate quantitative analysis models. It was found that the extreme learning machine could achieve good detecting performances with the overall accuracies of 99.20% and 89.38% for the laboratory-inoculated and field-infected samples, respectively [16]. Despite reasonable classification results by applying hyperspectral imaging for analyzing and detecting the quality and safety of crop seeds, it is still challenging to apply these laboratory-based techniques to the more practical applications due to the high cost of the system. Therefore, a low-cost imaging system that can obtain spectral signatures at some key wavelengths and feasible discriminant model are deeply needed to monitor agricultural plant seed quality.

To our knowledge, few studies have been carried out to detect rice false smut (RFS) in rice seed using a low-cost imaging system. Therefore, the main objective of this research was to develop a low-cost narrow band multispectral imaging system and establish a feasible classification model for detecting rice false smut (RFS). To achieve this goal, the specific objectives were to (1) develop a low-cost multispectral imaging system with six narrow bands distributed in the visible/near infrared (Vis/NIR) region; (2) acquire the multispectral images of healthy and RFS infected samples of two different cultivars of rice seeds; and (3) establish a feasible classification model for rice false smut (RFS) discrimination.

## 2. Materials and Methods

### 2.1. Rice Seeds and Pathogen Inoculation

**Figure 1:**
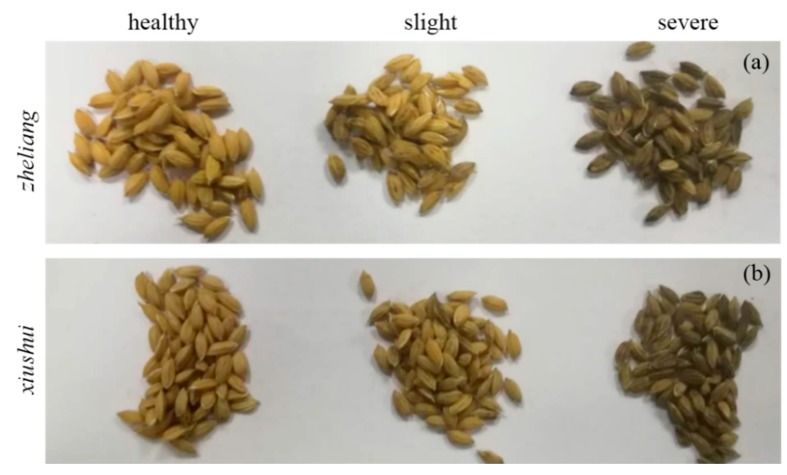
Two genotypes of rice seeds *Zheliang* (**a**) and *Xiushui* (**b**) with different infected degrees of rice false smut (RFS).

Two genotypes of rice plants including *Zheliang* and *Xiushui* that are susceptible to rice false smut (RFS) were used in this study. The rice plants and *Ustilaginoidea virens* strain were provided by the College of Biosystems Engineering and Food Science, Zhejiang University. In this study, the plants were inoculated with conidial suspensions according to previous research [17,18]. Briefly, the *Ustilaginoidea virens* strain was cultivated with potato sucrose broth (PS) in an incubator shaker for five days at 150 rpm. at 28 °C. For spore inoculation experiments, about 1.5 mL of the conidial suspension (2 × 10^5^ conidia mL^−1^) was injected into a single panicle using a syringe in the late afternoon at the booting stage, while the control plants were infected with double distilled water (ddH_2_O) according to previous study [18]. After inoculation, the rice plants were kept at 90% relative humidity and 25 °C for 48 h, subsequently at 98% relative humidity for an additional five days, and finally all plants (control and infected) were transferred to a green house. The rice seeds were collected 25 days later after inoculation, and were stored in textile bags in a storage room with a relative humidity of 65% and average temperature of 15 °C before image acquisition. Different disease severities were selected to produce enough infected stages of seeds with the aim of establishing a reliable classification model. Finally, 473 healthy, 349 slight, and 95 severe seeds of *Zheliang* and 392 healthy, 430 slight, and 186 severe seeds of *Xiushui* were selected for analysis (Figure 1). A significant difference could be observed between the healthy and severe infected rice seeds, while it did not present distinct differences between the healthy and slightly infected samples. It was hard to identify RFS in rice seed in the early phase only using information from the RGB images.

### 2.2. Multispectral Imaging System Design, Images Acquisition, and Processing

In order to collect multispectral images of rice seeds from visible to the near-infrared region for rice false smut (RFS) detection, a multispectral imaging system was developed in this study (Figure S1a,b). Some key components can significantly affect the outperformance of the system include the quantum efficiency (QE) of charge coupled device (CCD), spectrum peak of light-emitting diode (LED), and distribution of illumination so were taken into consideration. To obtain a high quality of spectral images in the near-infrared region, the QE of CCD in the near-infrared region should be enhanced. In this case, a monochrome CCD sensor (MT9V032, ON Semiconductor, Phoenix, AZ, USA), where the QE in near-infrared region was about 45% at 750 nm and 30% of QE at 850 nm, respectively, was selected. The focal length of the camera lens was 8 mm with a standard view (M0814-MP2, Tsukishima, Chao-ku, Tokyo, Japan). In order to collect spectral images at different wavelengths, six commercial narrow-band LEDs with central spectrum peaks at 460, 520, 660, 740, 850, and 940 nm with full width at half maximum of 25 nm, respectively, were selected to provide light sources in the range of the visible and near-infrared region (Epileds Technologies, Inc., Taiwan, China), which were easily bought from a website. The power of each LED was 3 W, which can provide enough lighting intensity for image collection. Two LEDs of each wavelength were symmetrically arrayed around the lens, and a total of 12 LEDs with an angle of 30° to each other were installed at the top of this system (Figure S1c). Twelve LED drivers (U7375, Xinhuakai Optoelectronics Co. Ltd., Shenzhen, China) were used to power the LEDs. Additionally, four mirrors were installed at four sides of the dark box to ensure that the light distribution was as homogeneous as possible. The total cost of the system hardware was less than 350 $. The multispectral images collected by CCD camera were controlled by a software developed by our group using the C++ programming language based on the platform Visual Studio 2018 (Microsoft, Redmond, WA, USA). During the process of multispectral image acquisition, rice seed samples were placed on the sample holder, and the distance between the lens and rice seeds was 18 cm. The exposure time of the CCD camera was 30 ms. The multispectral image preprocessing was first conducted on the original multispectral images for the correction using a flat-field (MFB99-50-17, Institute of Optics and Precision Machinery of Chinese Academy of Sciences, Anhui, China) based on the following equation:

*R* = (*I*_*raw*_ − *I*_*dark*_)/(*I*_*ref*_ − *I*_*dark*_) (1)

where *R, I_raw_, I_dark_*, and *I_ref_* were the corrected images, original images, dark current, and reference images, respectively. Mean reflectance spectra from the whole rice seed area (region of interest, ROI) was finally derived for further analysis. 

### 2.3. Principal Component Analysis (PCA)

In this study, the spectrum at different wavelengths was collected using a multispectral imaging system, and the individual reflectance can reflect the interaction between photons at specific wavelengths and plant materials. In order to ascertain the sensitivity of each wavelength to RFS, the principal component analysis (PCA) that used an orthogonal transformation was carried out by projecting the raw data into a new coordinate where the first component can describe the greatest variable of the original data on the first coordinate, the second greatest variance on the second coordinate, and so on [19]. After the application of PCA, the principal components were linearly uncorrelated, and the loadings (or coefficients) of each principal component was then used to qualitatively identify the importance of each wavelength that was related to rice false smut (RFS) infection.

### 2.4. Development and Validation of Classification Models for Rice False Smut (RFS) Detection

**Figure 2:**
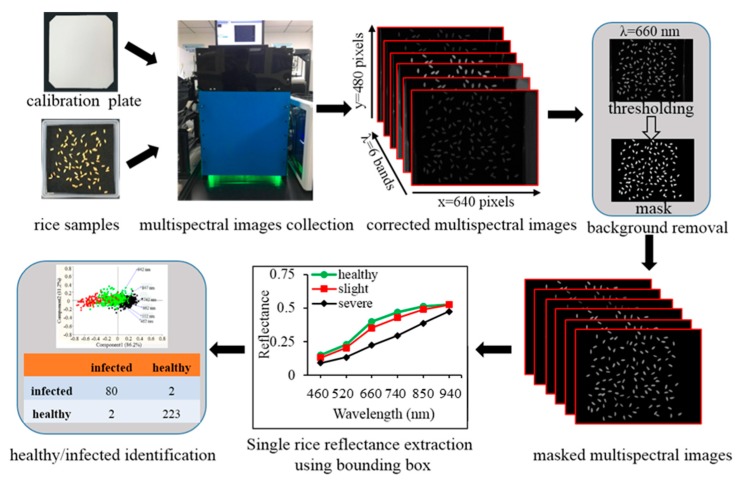
Schematic overview of the analytical procedure for rice false smut (RFS) disease detection.

In order to develop an optical classification model for discriminating rice false smut (RFS) infected rice seeds from the healthy ones, three commonly used classification models were introduced to classify spectral features extracted from spectral images at six wavelengths. The best model was finally selected according to the best classification performance. Least squares-support vector machine (LS-SVM) has been proven capable of addressing not only linear, but also nonlinear multivariate analysis problems with a relatively fast way of mapping the original data space to a hyper-plane space using a kernel function [20,21]. LS-SVM was modified from the standard SVM by employing a least squares linear cost function instead of quadratic programming to obtain the support vectors [22]. The LS-SVM also embodied a structural risk minimization principle to avoid overfitting. Before the application of LS-SVM for rice false smut (RFS) detection, two key parameters including regularization parameter (*γ*) and bandwidth (*δ^2^*) need to be optimized. The *γ* determined the tradeoff between minimizing the training error and model complexity, while the *δ^2^* was the bandwidth of the kernel function. In this study, the radial basis function (RBF) was selected, and the grid searching technique was introduced for optimizing the bandwidth (*δ^2^*) and regularization parameter (*γ*). Linear discriminant analysis (LDA) described a categorical variable by linearly combining a set of features that could interpret two or more classes of objects [23], which was also widely applied to the detection of diseased or damaged plant materials such as broken kernels identification in bulk wheat [24], chilling injury detection in jujube [25], and pathogen infestation detection in soybean [26]. In this study, LDA was also carried out to discriminate RFS infected rice seeds from normal ones in two different genotypes. Moreover, the K-nearest neighbor algorithm (KNN) was also used to detect RFS infected rice seeds. KNN is a non-parametric discriminant model through training k neighbors from the training set with a distance function. It classifies features into a specific class based on the largest voting rules [27]. In this study, four neighbors and Manhattan distance were decided to develop the KNN model. During the process of classification, the RFS infected and healthy rice seeds were labeled as “1” and “2”, respectively. Additionally, the Kennard–Stone (KS) algorithm was carried out to divide the whole data of each genotype into two groups with two thirds of the whole data for the training set and one third of whole data for the validation set [28]. The classification performances of the three different models were finally assessed based on the overall classification accuracy, false negative (FN), true negative (TN), true positive (TP), and false positive (FP) derived from the confusion matrix. With the aim to further validate the robustness of the RFS classification model developed from one cultivar to identify RFS in different rice cultivars, the strategy of model updating was implemented to rebuild the classification model by adding some representative samples into the original training dataset [29]. Data analysis in this study was implemented on the platform of MATLAB R2014a (MathWorks, Inc., Natick, MA, USA) and Excel 2011 (Microsoft, Redmond, WA, USA). The detailed procedure of RFS detection using multispectral imaging is shown in Figure 2. 

## 3. Results and Discussion

### 3.1. System Performance Assessment

**Figure 3:**
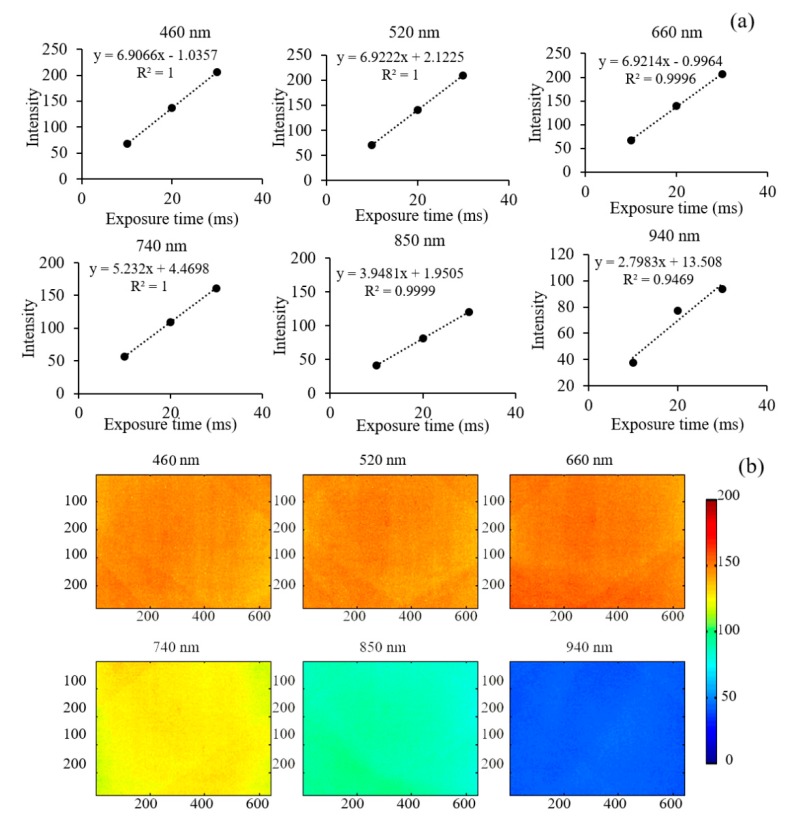
The linearities of CCD under different exposure time (**a**) and illuminance distribution (**b**) at wavelengths of 460, 520, 660, 740, 850, and 940 nm, respectively, at a working distance of 18 cm.

In order to understand the outperformance of CCD at six wavelengths, the linearities at 460, 520, 660, 740, 850, and 940 nm at a working distance of 18 cm were tested using a standard diffuse reference plate (MFB99-50-17, Institute of Optics and Precision Machinery of Chinese Academy of Sciences, Anhui, China), respectively, as shown in Figure 3a. It was found that there were good linearities with *R^2^* of 1.0000, 1.0000, 0.9996, 1.000, and 0.9999 for 460, 520, 660, 740, and 850 nm, respectively. Compared with linearities from these five wavelengths, it was relatively poor at 940 nm with a linearity of 0.9469. A good linearity of CCD can enable us to capture images using different exposure times of a CCD camera for the reference and samples with the aim of obtaining an optimal signal to noise (SNR).

Although original multispectral images can be corrected using a flat-field to eliminate the bias caused by the uneven distribution of light, it still needed to understand the illuminance distribution at every wavelength due to light from 12 LEDs emitting from different spatial positions (they were arrayed around the lens, Figure S1c). Figure 3b shows the illuminance distribution at 460, 520, 660, 740, 850, and 940 nm, respectively, at a working distance of 18 cm. It was noted that the illuminance distribution in the center was more uniform than that in the corner. The illuminance uniformity was defined as the standard deviation divided by the average illuminance, also named as the variable coefficient (CV). Therefore, the illuminance uniformity of the wavelength was 2.39%, 2.36%, 2.57%, 2.75%, 4.74%, and 3.18% for 460, 520, 660, 740, 850, and 940 nm, respectively. The CV values of the illuminance distribution at six wavelengths were all lower than 5%, indicting a uniform light distribution of this system.

### 3.2. Reflectance Spectra of Healthy and Rice False Smut (RFS) Infected Rice Seeds of Zheliang and Xiushui

**Figure 4:**
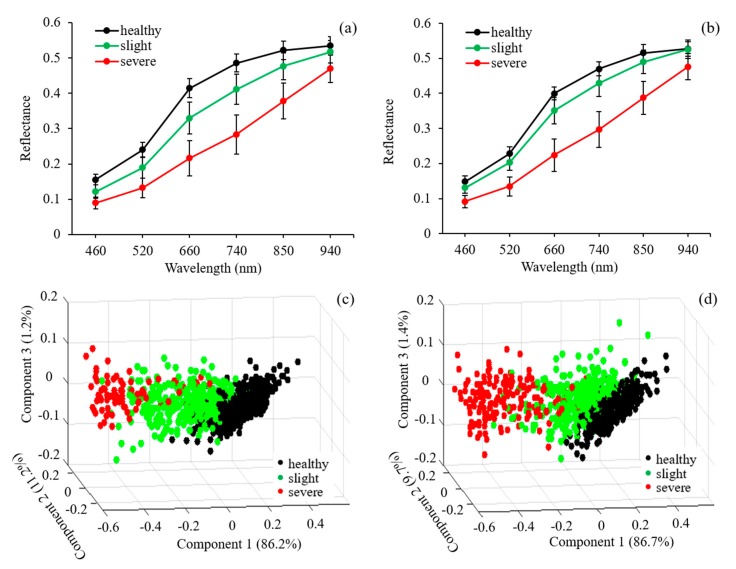
Mean reflectance spectra of healthy and rice false smut (RFS) infected rice seeds of *Zheliang* (**a**) and *Xiushui* (**b**). Principal component analysis of reflectance at six wavelengths in healthy, slightly, and severely infected rice seeds of *Zheliang* (**c**) and *Xiushui* (**d**).

Figure 4 shows the mean reflectance spectra at six wavelengths of healthy and rice false smut (RFS) infected rice seeds of *Zheliang* (Figure 4a) and *Xiushui* (Figure 4b) collected by the multispectral imaging system. A general reflectance pattern of rice seeds in the spectral region of 460–940 nm was observed from two cultivars with a higher reflectance in the NIR region than that in the visible range, which was similar with the previous research [30]. It could be observed that the pathogen decreased the reflectance both in the visible and near-infrared regions, and was reduced to a further extent with disease development. It presented a difference of reflectance between healthy and diseased rice seeds at 460, 520, and 660 nm, where it carried mostly the external features (i.e., color and/or surface defects) due to the fact that the pathogen can induce powdery dark green chlamydospore balls in the spikelets during the late phase of infection [18]. Compared with healthy rice seeds, reflectance changes of unhealthy rice seeds over the NIR range were mainly due to the changes in the internal chemical composition and tissue structure after pathogen infection. These different patterns of the reflectance spectra between healthy and diseased samples presented a possibility of identifying RFS infected rice seeds from healthy ones using this multispectral imaging system.

*Ustilaginoidea virens* caused changes in the quality of rice seeds that were reflected in the modification of the reflectance spectra at six wavelengths (Figure 4a,b). In order to figure out which wavelength was the most sensitive to pathogen infection, the principal component analysis (PCA) was then carried out by projecting reflectance at six wavelengths into a new coordinate where the first several components can express the most contribution of original data according to their impact on the total response of rice seeds to rice false smut disease. It can be observed that the first three components described about 98.6% of the total information in rice seeds of *Zheliang* with 86.2%, 11.2%, and 1.2% for component 1, component 2, and component 3, respectively (Figure 4c). For *Xiushui*, the first three components contributed to about 97.8% of total information with 86.7%, 9.7%, and 1.4% for component 1, component 2, and component 3, respectively (Figure 4d). Through the analysis from the 3D vector graphs (Figure 4c,d), a pattern was found where the samples were generally grouped into three groups in both cultivars depending on different degrees of disease, though some overlaps were found between the healthy and slightly infected samples. The overlaps between healthy and some slightly infected samples might be due to the fact that the surface features or inside the chemical component of the slightly infected seeds did not change too much. Additionally, the diseased samples were almost clustered in the left corner of the plane in comparison with the healthy ones. Compared with healthy rice seeds, it could be observed that the distribution of the diseased samples on the plane of vector graphs mainly resulted from the decreased value of component 1. Considering the difference in the value of component 2 among the healthy, slightly, and severely infected rice seeds, it did not present as significant as component 1 without obvious changes together with rice false smut (RFS) disease development.

The direction of each wavelength in the vector graph indicted the effect on component value, while magnitude could serve as the impact of rice false smut (RFS) disease on reflectance. Table 1 shows the sensitivity and contribution to the component value of each wavelength responding to false smut (RFS) disease infection in two cultivars. For principal component 1, the six wavelengths all contributed to increase its value in both two cultivars, which was in line with the results from Figure 3a,b, where *Ustilaginoidea virens* reduced the reflectance. Comparing the magnitudes among the six wavelengths, it could be observed that the reflectance at 740 nm was the most sensitive to *Ustilaginoidea virens* infection with the contribution coefficient of 0.431 and 0.433 for *Zheliang* and *Xiushui*, respectively. The sensitivity of each wavelength to false smut (RFS) disease for the two cultivars were similar with a descending order of 740, 660, 520, 850, 460, and 940 nm for *Zheliang* and 740, 660, 850, 520, 460, and 940 nm for *Xiushui*, respectively. The value of principal component 2 was mainly determined by 740, 850, and 940 nm with positive values and 460, 520, and 660 with negative values. 

### 3.3. Spectral Features for Rice False Smut (RFS) Detection in Rice Seeds

The performance of the three classification models using six reflectances at 460, 520, 660, 740, 850, and 940 nm collected from a multispectral imaging system as model inputs was evaluated. Rice false smut (RFS) detecting results from LDA, KNN, and LS-SVM are summarized in Table 2. It was found that the classification results varied using different classification models, indicating that it was of importance to select an optimal model for false smut (RFS) detection in rice seeds. For *Zheliang*, LDA and KNN achieved the same overall accuracy of 94.8%. Further analyzing the detection result of infected seeds from LDA and KNN, 85.4% of infected samples were correctly detected with the fact that 12 out of 82 infected samples were misclassified into the healthy class with a false negative rate of 14.6% for LDA, while 96.7% of infected samples were correctly detected with a false negative rate of 3.2% for KNN. Although the overall classification accuracy was the same between LDA and KNN, the KNN model achieved a relatively low false negative rate of the RFS detection, which is critical in finding as many infected samples as possible to prevent further infection. Compared with LDA and KNN, the classification accuracies for infected and healthy seeds of the LS-SVM model were in general better, as presented in Table 2. The classification accuracies for infected and healthy rice seeds were 97.6% and 99.1%, respectively. This achieved a 98.7% overall classification with the false negative rate of 3.2%. Considering the detection performance of RFS in *Xiushui*, the overall classification accuracies for LDA, KNN, and LS-SVM were in general poorer than those in *Zheliang*, which was reasonable because the disease in *Xiushui* was less severe, as shown in Figure 4b. The LS-SVM classifier also obtained the best result for *Xiushui* in comparison with the LDA and KNN with the overall classification accuracy of 91.4% and false negative rate of 6.7%, respectively. These results imply that the LS-SVM was feasible for rice false smut (RFS) disease detection in rice seeds and the spectral resolution at these six narrow bands was enough to collect different information between healthy and RFS infected rice seed. It also needs to be mentioned that the classification accuracies for two different cultivars achieved in this study prove the feasibility of this multispectral imaging system for rice false smut (RFS) disease detection.

### 3.4. Validation of the Classification Model Using Model Transfer 

**Figure 5:**
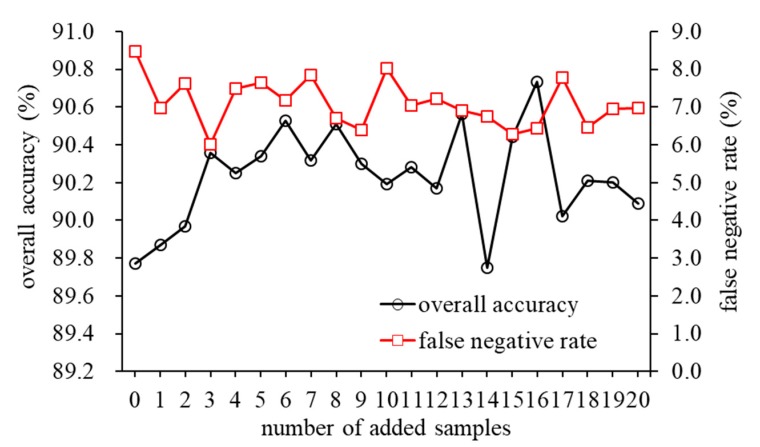
The overall accuracies and false negative rates from the least squares-support vector machine (LS-SVM) for rice false smut (RFS) disease detection in *Xiushui* based on the model established from *Zheliang*.

In this study, the feasibility of detecting rice false smut (RFS) using multispectral imaging in two cultivars of *Zheliang* and *Xiushui* based on the LS-SVM model with the corresponding training dataset was proven, as shown in Table 2. Generally, it is a robust model for RFS detection that is meant to be applicable for different cultivars of rice seed. An alternative is to apply the chemometrics technique of model updating to correct the differences caused by cultivar variation, thereby making the classification model transferable, avoiding the substantial cost and time to rebuild a new model. As shown in Table 2, the LS-SVM achieved a better detection performance of RFS in *Zheliang* than that in *Xiushui*. Therefore, we used data from *Zheliang* to train the LS-SVM model to identify RFS in *Xiushui*. In this study, the LS-SVM model from *Zheliang* was updated by adding a few samples from *Xiushui*. Figure 5 displays the overall accuracies and false negative rates of rice false smut (RFS) disease detection in *Xiushui*, based on the model established from *Zheliang*, with the addition of different new samples from *Xiushui* selected by the Kennard–Stone (KS) algorithm. There was an increasing pattern of overall accuracies when the added samples increased from 1 to 3, and became relatively stable in the range of 4–8, indicating that the combination of two thirds of the whole *Zheliang* data with eight samples from *Xiushui* could provide enough variance about *Xiushui* in the new model. In this circumstance, the LS-SVM model with the values of *γ* and *σ^2^* of 7748.7 and 0.04, respectively, was considered practically for RFS detection that could span both *Zheliang* and *Xiushui*. It obtained an overall accuracy of 90.3% with a false negative rate of 7.8% during the process of RFS detection in *Xiushui*, which was comparable to those from the model using its own data as the training set, as shown in Table 2 (the overall accuracy and false negative rate was 91.4% and 6.7%, respectively). These results not only underline the robustness of the LS-SVM model established from *Zheliang* for RFS detection in different cultivars of rice seed, but also demonstrate that the availability of the developed multispectral imaging system only using six wavebands still achieved a good classification performance of RFS detection in two cultivars of rice seeds.

## 4. Conclusions

In this study, we demonstrated the feasibility of a low-cost multispectral imaging system combined with chemometrics for rice false smut (RFS) detection in two different cultivars. Multispectral images at 460, 520, 660, 740, 850, and 940 nm yielded a significant difference between the healthy and RFS infected rice seeds. The LS-SVM model established from six reflectances could be used for discriminating RFS infected rice seeds from healthy ones with the overall classification and the false negative rate of 98.7% and 3.2% for *Zheliang*, respectively, and 91.4% and 6.7% for *Xiushui*, respectively, Additionally, the RFS discriminant model developed by *Zheliang* was successfully transferred to *Xiushui* with an overall accuracy of 90.3% and false negative rate of 7.8%. Overall, the multispectral imaging system developed in this study, combined with the LS-SVM model, can be implemented for fast, on-line RFS detection in rice seeds.

## Figures and Tables

**Table 1:** Contribution of changes in the reflectance at six wavelengths into total variation of principal components in rice seeds of *Zheliang* and *Xiushui.*

Wavelengths (nm)	*Zheliang*		*Xiushui*	
	Component 1	Component 2	Component 1	Component 2
460 nm	0.400	−0.456	0.397	−0.473
520 nm	0.419	−0.353	0.419	−0.357
660 nm	0.431	−0.157	0.427	−0.134
740 nm	0.433	0.028	0.431	0.043
850 nm	0.417	0.322	0.421	0.275
940 nm	0.343	0.734	0.349	0.744

**Table 2:** Classification accuracies of rice seeds of *Zheliang* and *Xiushui* based on the spectral features from different classification models.

Cultivars		*Zheliang*			*Xiushui*		
Models	Predicted Class	Actual Class					
		Infected	Healthy	Accuracy (%)	Infected	Healthy	Accuracy (%)
LDA	infected	70	12	85.4	150	28	84.3
	healthy	4	221	98.2	8	150	94.9
Overall accuracy (%)		94.8			89.3		
KNN	infected	80	2	97.6	170	8	95.5
	healthy	14	211	93.8	36	122	77.2
Overall accuracies (%)		94.8			86.9		
LS-SVM *	infected	80	2	97.6	166	12	93.3
	healthy	2	223	99.1	17	141	89.2
Overall accuracy (%)		98.7			91.4		

* Regularization parameter (*γ*) and bandwidth (*σ^2^*) of LS-SVM was 456894.20 and 0.46 for *Zheliang*, and 5911.85 and 4.15 for *Xiushui*.

## Supplementary Materials

The following are available online at https://www.mdpi.com/1424-8220/20/4/1209/s1, Figure S1. The multispectral imaging system (MSI) developed in this study. (a) and (b) were the overview and details of MSI. (c) was the upward view of 12 narrow-band LEDs arrayed around the camera lens.
