# Supplementary material for: Development of a Low-Cost Narrow Band Multispectral Imaging System Coupled with Chemometric Analysis for Rapid Detection of Rice False Smut in Rice Seed
Source: Sensors (Basel). 2020 Feb 22;20(4):1209. doi: 10.3390/s20041209 (PMC7070825; doi:10.3390/s20041209)
Supplement: Supplementary file 1 [file sensors-20-01209-s001.pdf]

Development of a Low-cost Narrow Band Multispectral Imaging System Coupled with Chemometric Analysis for Rapid Detection of Rice False Smut in Rice Seed

Haiyong Weng[1], Ya Tian[1], Na Wu[2], Xiaoling Li[3], Biyun Yang[1], Yiping Huang[1], Dapeng Ye[1,*] and Renye Wu[4,*]

[1]College of Mechanical and Electrical Engineering, Fujian Agriculture and Forestry University, Fuzhou 310002, P.R. China

[2]College of Biosystems Engineering and Food Science, Zhejiang University, Hangzhou 310058, P.R. China

[3]College of Mechanical Engineering and Automation, Fuzhou University, Fuzhou 310002, P.R. China

[4] College of Agriculture, Fujian Agriculture and Forestry University, Fuzhou 310002, P.R. China

* Correspondence: ydp@fafu.edu.cn; renye.wu@fafu.edu.cn

Full list of author information:

Haiyong Weng: hyweng@zju.edu.cn; Ya Tian: 13133969161@163.com; Na Wu: nawu018@zju.edu.cn; Xiaoling Li: xiaoling_fzu@163.com; Biyun Yang: yangbiyun2010@126.com; Yiping Huang: wing361686@gmail.com; Dapeng Ye: ydp@fafu.edu.cn; Renye Wu : renye.wu@fafu.edu.cn

A multispectral imaging system was developed for rice false smut (RFS) detection. It collected six spectral images at spectrum peaks of 460, 520, 660, 740, 850 and 940 nm, respectively, using a monochrome CCD sensor (MT9V032, ON Semiconductor, Phoenix, Arizona, USA). The light source was provided by 12 narrow-band high-power LEDs (Epileds Technologies, Inc., Taiwan, China). Four mirrors were installed at four sides of the dark box to ensure the light distribution as homogeneous as possible. The multispectral images data acquisition were performed on a software developed by our group using C++ programming language based on the platform Visual Studio 2018 (Microsoft, State of Washington, USA).

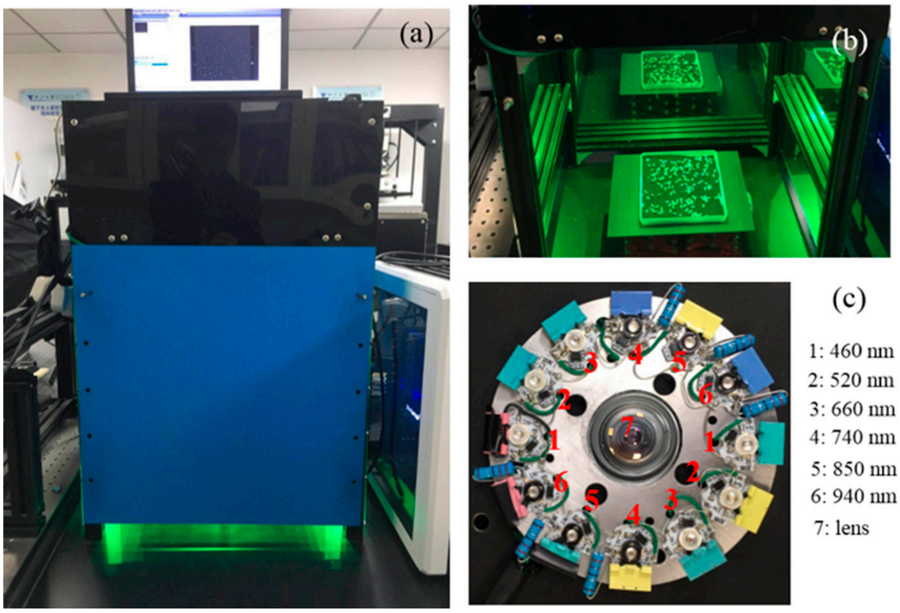

Figure S1. The multispectral imaging system (MSI) developed in this study. (a) and (b) were the overview and details of MSI. (c) was the upward view of 12 narrow-band LEDs arrayed around the camera lens.
